# Twice-Daily Dosing of Dolutegravir in Infants on Rifampicin Treatment: A Pharmacokinetic Substudy of the EMPIRICAL Trial

**DOI:** 10.1093/cid/ciad656

**Published:** 2023-10-26

**Authors:** Tom G Jacobs, Vivian Mumbiro, Uneisse Cassia, Kevin Zimba, Damalie Nalwanga, Alvaro Ballesteros, Sara Domínguez-Rodríguez, Alfredo Tagarro, Lola Madrid, Constantine Mutata, Moses Chitsamatanga, Mutsa Bwakura-Dangarembizi, Alfeu Passanduca, W Chris Buck, Bwendo Nduna, Chishala Chabala, Elizabeth Najjingo, Victor Musiime, Cinta Moraleda, Angela Colbers, Hilda A Mujuru, Pablo Rojo, David M Burger, EMPIRICAL Clinical Trial Group, Jahit Sacarlal, Muhammad Sidat, Elias Manjate, Sónia Martins, Stella Langa, Natália Nipaco, Sara Machava, Anastância Chirindza, Luzidina Martins, Mércia Nhaca, Kusum J Nathoo, Moses Chitsamatanga, Ruth Marange, Shepherd Mudzingwa, Dorothy Murungu, Natasha Namuziya, Idah Zulu, Perfect Shankalala, Mulima Mukubesa, Juliet Namwinwa, Chalwe Chibuye, Terence Chipoya, Veronica Mulenga, Bwalya Simunyola, John Tembo, Muleya Inambao, Salome Chitondo, Wyclef Mumba, Endreen Mankushe, Henry Musukwa, Davies Sondashi, Albert Kamugisha, Karen Econi, Andrew Kiggwe, Judith Beinomugisha, Sharafat Nkinzi, Lawrence Kakooza, Henriator Namisanvu, Nancy Lajara Mark, Josam Thembo Mwesige, Ivan Segawa, Joseph Ssessanga, Paul Mbavu, Bosco Kafufu, Denis Nansera, Elizabeth Najjingo, Bashira T Mbabazi, Abbas Lugemwa, Mariam Kasozi, Rogers Ankunda, Lilit Manukyan

**Affiliations:** Department of Pharmacy, Radboudumc Institute for Medical Innovation, Radboud University Medical Center, Nijmegen, The Netherlands; University of Zimbabwe Clinical Research Centre, Harare, Zimbabwe; Universidade Eduardo Mondlane Faculdade de Medicina, Maputo, Mozambique; University Teaching Hospitals-Children’s Hospital, Lusaka, Zambia; Department of Paediatrics and Child Health, School of Medicine, College of Health Sciences, Makerere University, Kampala, Uganda; Pediatric Unit for Research and Clinical Trials, Hospital 12 de Octubre Health Research Institute, Biomedical Foundation of Hospital Universitario 12 de Octubre, Madrid, Spain; Pediatric Unit for Research and Clinical Trials, Hospital 12 de Octubre Health Research Institute, Biomedical Foundation of Hospital Universitario 12 de Octubre, Madrid, Spain; Pediatric Unit for Research and Clinical Trials, Hospital 12 de Octubre Health Research Institute, Biomedical Foundation of Hospital Universitario 12 de Octubre, Madrid, Spain; Pediatric Service, Infanta Sofia University Hospital, Servicio Madrileño de Salud, Madrid, Spain; Universidad Europea de Madrid, Madrid, Spain; Pediatric Unit for Research and Clinical Trials, Hospital 12 de Octubre Health Research Institute, Biomedical Foundation of Hospital Universitario 12 de Octubre, Madrid, Spain; London School of Hygiene and Tropical Medicine, London, United Kingdom; University of Zimbabwe Clinical Research Centre, Harare, Zimbabwe; University of Zimbabwe Clinical Research Centre, Harare, Zimbabwe; University of Zimbabwe Clinical Research Centre, Harare, Zimbabwe; Universidade Eduardo Mondlane Faculdade de Medicina, Maputo, Mozambique; Universidade Eduardo Mondlane Faculdade de Medicina, Maputo, Mozambique; David Geffen School of Medicine, University of California–Los Angeles, Los Angeles, California, USA; Arthur Davidson Children’s Hospital, Ndola, Zambia; University Teaching Hospitals-Children’s Hospital, Lusaka, Zambia; School of Medicine, University of Zambia, Lusaka, Zambia; HerpeZ, Lusaka, Zambia; Mbarara Regional Referral Hospital, Mbarara, Uganda; Department of Paediatrics and Child Health, School of Medicine, College of Health Sciences, Makerere University, Kampala, Uganda; Joint Clinical Research Centre, Kampala, Uganda; Pediatric Unit for Research and Clinical Trials, Hospital 12 de Octubre Health Research Institute, Biomedical Foundation of Hospital Universitario 12 de Octubre, Madrid, Spain; Pediatric Service, Hospital Universitario 12 de Octubre, Servicio Madrileño de Salud, Madrid, Spain; Department of Pharmacy, Radboudumc Institute for Medical Innovation, Radboud University Medical Center, Nijmegen, The Netherlands; University of Zimbabwe Clinical Research Centre, Harare, Zimbabwe; Pediatric Unit for Research and Clinical Trials, Hospital 12 de Octubre Health Research Institute, Biomedical Foundation of Hospital Universitario 12 de Octubre, Madrid, Spain; Pediatric Service, Hospital Universitario 12 de Octubre, Servicio Madrileño de Salud, Madrid, Spain; Complutense University of Madrid, Madrid, Spain; Department of Pharmacy, Radboudumc Institute for Medical Innovation, Radboud University Medical Center, Nijmegen, The Netherlands

**Keywords:** dolutegravir, rifampicin, drug–drug interaction, infants, HIV

## Abstract

**Background:**

We evaluated dolutegravir pharmacokinetics in infants with human immunodeficiency virus (HIV) receiving dolutegravir twice daily (BID) with rifampicin-based tuberculosis (TB) treatment compared with once daily (OD) without rifampicin.

**Methods:**

Infants with HIV aged 1–12 months, weighing ≥3 kg, and receiving dolutegravir BID with rifampicin or OD without rifampicin were eligible. Six blood samples were taken over 12 (BID) or 24 hours (OD). Dolutegravir pharmacokinetic parameters, HIV viral load (VL) data, and adverse events (AEs) were reported.

**Results:**

Twenty-seven of 30 enrolled infants had evaluable pharmacokinetic curves. The median (interquartile range) age was 7.1 months (6.1–9.9), weight was 6.3 kg (5.6–7.2), 21 (78%) received rifampicin, and 11 (41%) were female. Geometric mean ratios comparing dolutegravir BID with rifampicin versus OD without rifampicin were area under curve (AUC)_0–24h_ 0.91 (95% confidence interval, .59–1.42), C_trough_ 0.95 (0.57–1.59), C_max_ 0.87 (0.57–1.33). One infant (5%) receiving rifampicin versus none without rifampicin had dolutegravir C_trough_ <0.32 mg/L, and none had C_trough_ <0.064 mg/L. The dolutegravir metabolic ratio (dolutegravir-glucuronide AUC/dolutegravir AUC) was 2.3-fold higher in combination with rifampicin versus without rifampicin. Five of 82 reported AEs were possibly related to rifampicin or dolutegravir and resolved without treatment discontinuation. Upon TB treatment completion, HIV viral load was <1000 copies/mL in 76% and 100% of infants and undetectable in 35% and 20% of infants with and without rifampicin, respectively.

**Conclusions:**

Dolutegravir BID in infants receiving rifampicin resulted in adequate dolutegravir exposure, supporting this treatment approach for infants with HIV–TB coinfection.

## Teaser

Dolutegravir trough concentrations in plasma were adequate for infants with human immunodeficiency virus receiving dolutegravir twice daily with rifampicin following World Health Organization weight-band dosing recommendations and comparable to trough concentrations in infants receiving dolutegravir once daily without rifampicin.

Approximately 50 000 children with human immunodeficiency virus (HIV, CWH) develop tuberculosis (TB) annually, and TB accounts for around 20% of total pediatric HIV-related deaths [1]. Despite receiving adequate antiretroviral therapy (ART), CWH are at higher risk of developing TB and tend to experience more rapid disease progression compared with their peers without HIV [2]. Furthermore, TB is most deadly for CWH during the first years of life [3]. Hence, it is essential to effectively treat both infections simultaneously in children with HIV-associated TB, which is complicated by adherence issues, overlapping toxicities, risk of immune reconstitution inflammatory syndrome, and drug–drug interactions (DDIs) between ART and TB treatment [4].

Recently, dolutegravir-based ART regimens have been established as the preferred first-line and second-line treatment options for children who are aged at least 4 weeks and weigh more than 3 kg [5–7]. Dolutegravir is primarily metabolized by uridine glucuronosyltransferase (UGT) 1A1 into its inactive metabolite dolutegravir-glucuronide and, to a lesser extent, by cytochrome P450 3A4 (CYP3A4) and UGT1A9 [8]. Rifampicin, an essential component of TB treatment, interacts substantially with dolutegravir by increasing dolutegravir metabolism through induction of UGT1A1, UGT1A9, and CYP3A4, resulting in a reduction in dolutegravir area under the plasma concentration-time curve (AUC), maximum concentration (C_max_), and trough concentration (C_trough_) by 54%, 43%, and 72% in healthy adults, respectively [9]. Adapting the dolutegravir dosing interval from once daily (OD) to twice daily (BID) was safe and effective in both adults and children [10, 11]. However, only limited data were available for children on dolutegravir dispersible tablets (DTs), and no pharmacokinetic data were available for infants who weighed less than 14 kg in whom the maturation of metabolic enzyme activity may not yet be fully complete [11, 12]. Ontogeny of UGT1A1 primarily occurs within the first 3–6 months of life, whereas CYP3A4 reaches adult levels in children aged between 1 and 5 years [13]. To this end, the magnitude of DDIs may be different in infants and young children compared with older children [4]. Furthermore, the specific contributions of UGT1A1 and CYP3A4 induction to increased metabolism of dolutegravir and whether these contributions change with age as metabolic enzymes mature remain uncertain. Investigating the metabolic ratio of dolutegravir plasma exposure to its metabolite could clarify this issue [14].

Lacking data in infants, combined with the rapidly increasing use of dolutegravir among infants that resulted from the global rollout of dispersible dolutegravir tablets and the absence of adequate alternative treatment options, stresses the urgent need for pharmacokinetic data in infants receiving dolutegravir with rifampicin [4, 6]. Our aim in this study was to evaluate dolutegravir pharmacokinetics in infants with HIV receiving dolutegravir BID with concomitant rifampicin, following World Health Organization (WHO) weight-band dosing.

## METHODS

### Study Design and Participants

This was a 2-arm, open-label, nonrandomized, multicenter pharmacokinetic substudy with descriptive safety and efficacy analysis of the EMPIRICAL randomized, controlled trial (NCT03915366) that aims to determine whether empirical treatment for cytomegalovirus and TB improves the survival of infants with HIV who are admitted with severe pneumonia [15]. The main trial includes infants aged 28–365 days with HIV and pneumonia who met the criteria for hospitalization and parenteral antibiotics following WHO guidelines [15]. Eligible infants in the main trial were first randomized to rifampicin-based TB treatment plus the standard of care (SOC; intravenous antibiotics, therapeutic cotrimoxazole, and prednisolone for the treatment of *Pneumocystis jirovecii* pneumonia) or to SOC alone. The second randomization was to valganciclovir for 15 days plus SOC versus SOC alone. If a clinical or laboratory diagnosis of TB was made in an infant not randomized to TB treatment, rifampicin-based TB treatment was initiated [15]. Consequently, we anticipated enrolling a larger number of infants on rifampicin compared with infants without rifampicin for this study.

This pharmacokinetic substudy recruited infants who weighed more than 3 kg at the time of pharmacokinetic sampling and were receiving dolutegravir BID with rifampicin compared with OD without rifampicin-based TB treatment from hospitals in Mozambique, Uganda, Zambia, and Zimbabwe. All infants needed to be on dolutegravir treatment for at least 14 days and rifampicin for at least 30 days. Exclusion criteria for this substudy included the use of concomitant medications known to have DDIs with dolutegravir, grade 4 anemia or likelihood of progressing to grade 4 anemia at the day of sampling, and vomiting within 4 hours of drug administration [16]. The EMPIRICAL trial protocol, including the pharmacokinetic substudies, was approved by local ethics committees and national ethical and regulatory authorities. Written informed consent was obtained from the caregivers of the infants, with the consent documents translated into local languages.

### Procedures

All infants received dolutegravir 10 mg scored DTs with abacavir/lamivudine, following national guidelines. Dosing of dolutegravir followed the WHO weight bands; children weighing 3 to <6 kg and 6 to <10 kg were administered 0.5 and 1.5 dolutegravir 10 mg DTs, respectively, with the dose frequency increased from OD to BID while on rifampicin [6]. Furthermore, rifampicin was given as part of a fixed-dose DT (rifampicin/isoniazid/pyrazinamide 75/50/150 mg) with ethambutol 100 mg DTs and dosed in accordance with the WHO pediatric dosing guidance; infants weighing 4 to 7 kg and 8 to 11 kg received 75 mg and 150 mg rifampicin, respectively [17].

Six blood samples were collected from each participant over 12 (BID) or 24 (OD) hours at predetermined intervals (predose and at 2, 4, 6, 8, and 12/24 hours after drug administration) within 30–60 days after enrollment in the main trial. The volume of blood collected from each participant did not exceed the maximum limit of 2.5% of the total blood volume for sick children [18]. Infants were considered fed if they received food or breastmilk within 2 hours before or 1 hour after taking dolutegravir. Treatment adherence to both ART and TB treatment of each infant was recorded by their caretaker over 3 days prior to the pharmacokinetic sampling.

HIV viral load (VL) and safety data were obtained as part of the EMPIRICAL trial. All adverse events (AEs) and severe AEs (SAEs) reported within the first 180 days after enrollment in the trial were descriptively reported for all pharmacokinetic participants. Furthermore, virological outcomes, expressed as the proportion of infants with an undetectable VL and those with >1000 copies/mL on study visit day 180 (upon completion of the TB treatment course), were described for infants in both study groups who had received at least 120 days of dolutegravir-based ART.

Dolutegravir and dolutegravir-glucuronide plasma concentrations were quantified at the Pharmacy Department of the Radboud University Medical Centre in Nijmegen, the Netherlands, a laboratory that participates in an international quality control program for monitoring antiretroviral drugs, including dolutegravir [19]. The liquid chromatography-tandem mass spectrometry assay had a lower limit of quantification (LLOQ) of 0.05 mg/L for dolutegravir and 0.005 mg/L for dolutegravir-glucuronide [14]. The precision of the assay, expressed as coefficient of variation, showed a range of 1.9% to 7.7% within runs and 3.2% to 7.6% between runs. The accuracy of the assay was within 90.0% to 106.0%.

### Statistical Analyses

Dolutegravir and dolutegravir-glucuronide pharmacokinetic parameters were determined with noncompartmental pharmacokinetic analysis using WinNonlin (Phoenix 64 v8.3, Certara) and were described as geometric mean with an associated coefficient of variance. Similar to previous studies, pharmacokinetic profiles were considered nonevaluable if the predose minimum concentration of dolutegravir was more than 15 times lower than the end-of-dose interval C_trough_, suggesting potential nonadherence [11, 20, 21]. The first concentration below the LLOQ at the end of the curve was set to half the LLOQ. Subsequent concentrations below the LLOQ were recorded as undetectable.

The C_max_ and time to reach maximum concentration (T_max_) were directly derived from the plasma concentration-time curve. The AUC was calculated using the linear up-log down trapezoidal rule and oral clearance (CL/F) by dividing the dose by AUC. The AUC_0–24h_ for BID dosing was estimated by multiplying the AUC_0–12h_ by 2 to enable comparison with the AUC_0–24h_ for OD dosing. The apparent elimination half-life (T_1/2_) was calculated as 0.693 divided by the apparent terminal-phase elimination rate constant (λz), which was estimated through linear regression using logarithmic data for the last 3 data points of concentration versus time. Geometric mean ratios (GMRs) comparing dolutegravir AUC_0–24h_, C_max_, and C_trough_ for infants receiving rifampicin versus those without rifampicin were calculated using an unpaired *t* test on log-transformed data. The proportion of infants with a dolutegravir C_trough_ below the 90% effective concentration (EC_90_; 0.32 mg/L), the dolutegravir concentration at which 90% of the maximal VL reduction was achieved in a 10-day monotherapy study in adults, as well as below the in vitro protein-adjusted 90% maximal inhibitory concentration (IC_90_; 0.064 mg/L) was reported per study arm [22, 23]. The individual dolutegravir metabolic ratio was calculated by dividing the AUC_0–24h_ of dolutegravir-glucuronide by the AUC_0–24h_ of dolutegravir, after adjusting for the molar mass.

Correlation between dolutegravir AUC_0–24h_ and dolutegravir metabolic ratio with age and weight-for-height was evaluated using Spearman’s rank correlation method, separately for the 2 study groups. The statistical analyses were performed using IBM SPSS Statistics software (v27).

## RESULTS

### Demographics

**Figure 1.**
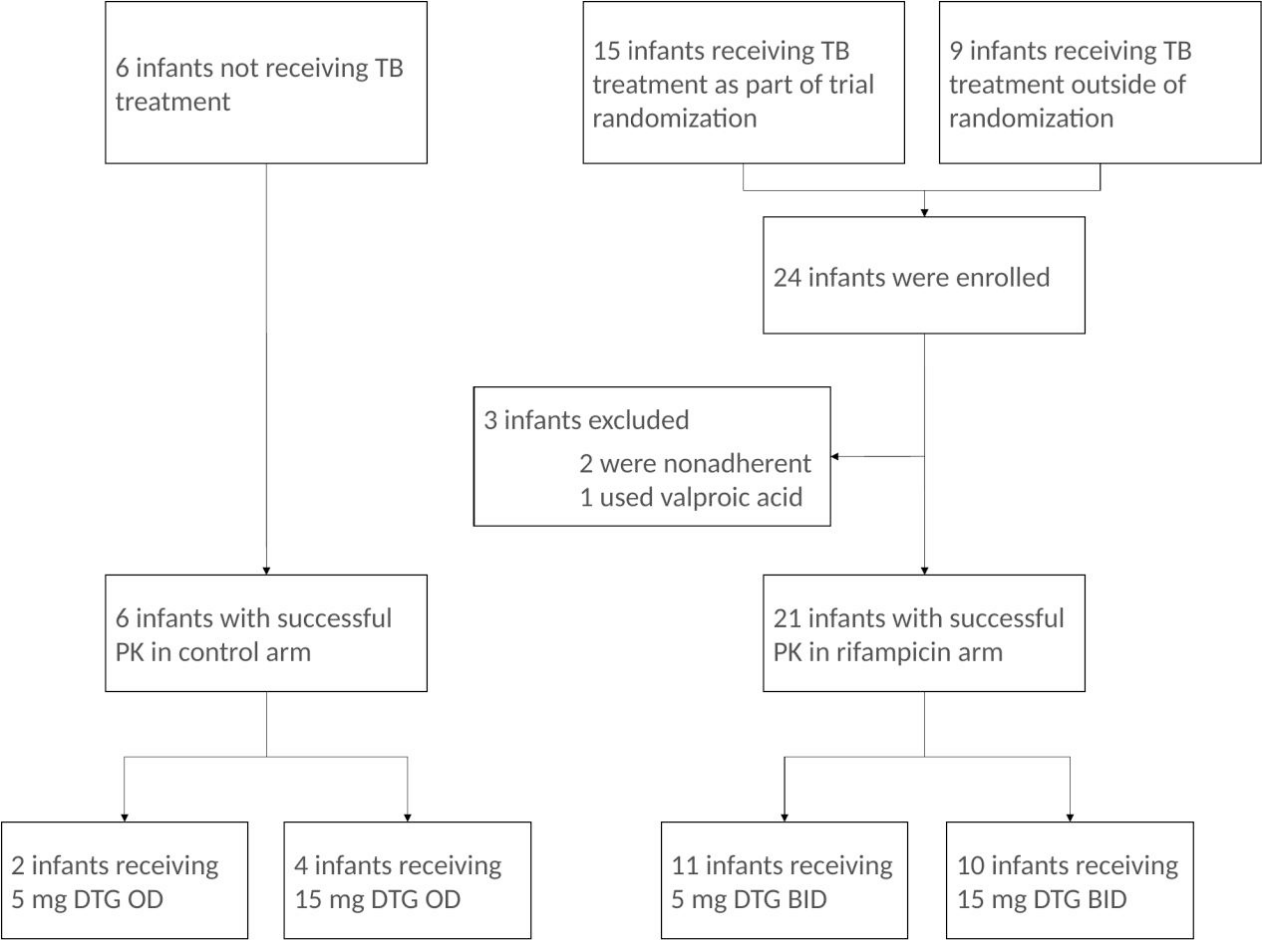
Study profile. Abbreviations: BID, twice-daily dosing; DTG, dolutegravir; OD, once-daily dosing; PK, pharmacokinetics; TB, tuberculosis.

A total of 30 infants were recruited between August 2021 and August 2022 (see Figure 1 for the study profile). Of these, 27 had evaluable pharmacokinetic profiles with a median (interquartile range) age of 7.1 months (6.1–9.9), weighing 6.3 kg (5.6–7.2), and 11 of 27 (41%) were female. Nonevaluable pharmacokinetic profiles were the result of nonadherence (n = 2) and comedication interaction with valproic acid (n = 1). Twenty-five of the 27 infants were considered to have been fed during the administration of dolutegravir at the pharmacokinetic visit. Demographic characteristics of the included infants are displayed in Table 1. One child was lost to follow-up; all other children completed study visit day 180 of the main trial.

**Table 1.** Demographics of Study Infants

Demographic	Pharmacokinetics and Safety Population	
	Control Arm (n = 6)	Rifampicin Arm (n = 21)
Male/Female	3/3	13/8
Weight, kg^a^	6.0 (5.8 to 8.0)	6.4 (5.2 to 7.1)
Weight-band, kg		
3–<6	3 (50%)	11 (52%)
6–<10	3 (50%)	10 (48%)
Length, cm^a^	64.5 (60.8 to 66.3)	62.0 (58.5 to 67.0)
Age, mo^a^	7.4 (6.4 to 8.9)	6.6 (5.6 to 10.5)
Weight-for-height *z* score^a^	−0.8 (−2.1 to 0.5)	−0.7 (−1.7 to 0.5)
Weight-for-age *z* score^a^	−2.4 (−3.0 to -0.5)	−2.5 (−4.4 to −0.9)
Baseline viral load, log_10_ copies/mL^a^	5.6 (4.0 to 6.6)	6.0 (5.6 to 6.8)
Treatment naive upon enrollment in the main trial	5 (83%)	19 (90%)
Dolutegravir dose mg/kg/24 h^a,b^	1.86 (1.09 to 2.36)	2.66 (2.00 to 4.16)
Confirmed tuberculosis diagnosis^c^		
Yes	0 (0%)	6 (29%)
No	6 (100%)	15 (71%)

Demographic data represent the infant on the day of their pharmacokinetic visit unless specified otherwise.

^a^Reported median (interquartile range).

^b^Infants in the rifampicin arm received dolutegravir twice daily and hence received a 2-times higher mg/kg/24 hour dose compared with the control arm using dolutegravir once daily.

^c^Diagnosed using GeneXpert and/or tuberculosis urine lipoarabinomannan.

### Pharmacokinetic Analyses

**Figure 2.**
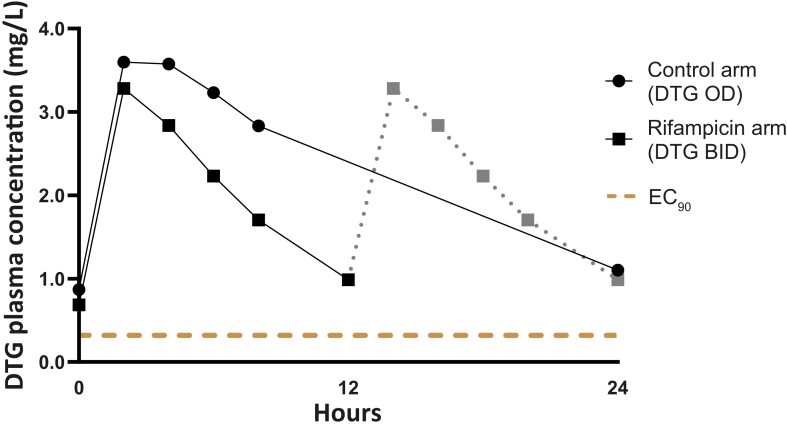
Geometric mean DTG concentration-time profiles for infants receiving rifampicin and the control arm. The solid lines in the BID curve represent observed concentrations; the gray dotted line represents imputed values by repeating the first 12-hour curve. Abbreviations: BID, twice-daily dosing; DTG, dolutegravir; EC_90_: 90% effective concentration; OD, once-daily dosing.

**Figure 3.**
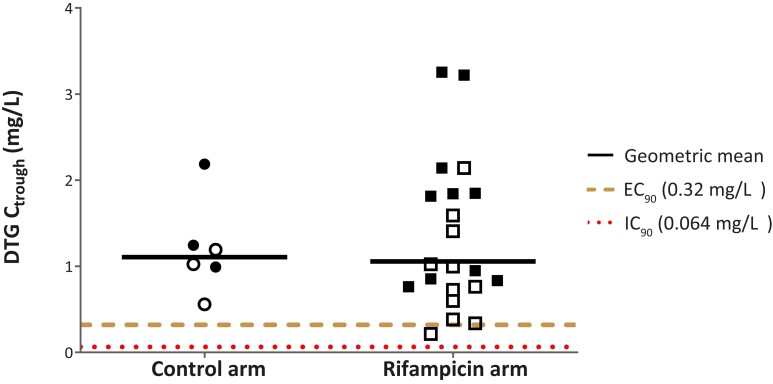
DTG trough concentrations in infants given once-daily DTG dosing without rifampicin (control arm) or twice-daily dosing with rifampicin. Open dots and squares represent infants receiving 5 mg (3 to <6 kg weight band); solid dots and squares indicate infants receiving 15 mg (6 to <10 kg weight band) DTG. Abbreviations: C_trough_, trough concentration; DTG, dolutegravir; EC_90_, 90% effective concentration; IC_90_, in vitro protein-adjusted 90% maximal inhibitory concentration.

The dolutegravir plasma concentration-time curves for the 2 study groups are displayed in Figure 2. One of 21 infants receiving rifampicin and none of 6 infants without rifampicin had a dolutegravir C_trough_ below the EC_90_ target (dolutegravir, 0.32 mg/L), and none of the participants had a C_trough_ below the IC_90_ target (dolutegravir, 0.064 mg/L), as shown in Figure 3 and Table 2. The dolutegravir GMRs comparing dolutegravir BID with rifampicin with dolutegravir OD without rifampicin were 0.91 (95% confidence interval [CI], .59–1.42) for AUC_0–24h_, 0.95 (95% CI, .57–1.59) for C_trough_, and 0.87 (95% CI, .57–1.33) for C_max_. The apparent oral clearance of dolutegravir was 2.1 times higher in infants receiving rifampicin compared with those not receiving rifampicin, and the metabolic ratio (dolutegravir-glucuronide/dolutegravir ratio) was 2.3 times higher (as shown in Table 2). No significant correlation was found between dolutegravir AUC_0–24h_ and dolutegravir metabolic ratio with age (*P* = .124 and *P* = .183, respectively) or weight-for-height (*P* = .747 and *P* = .297, respectively) (see Supplementary Figure 1).

**Table 2.** Dolutegravir Pharmacokinetic Parameters

Pharmacokinetic Parameter	Control Arm (n = 6)	Rifampicin Arm (n = 21)
Infants with C_trough_ <0.32 mg/L	0 (0%)	1 (5%)
Infants with C_trough_ <0.064 mg/L	0 (0%)	0 (0%)
Trough concentration, mg/L	1.11 (46.3%)	1.06 (82.4%)
Area under the plasma concentration-time curve over 24 h, h × mg/L	54.42 (39.0%)	49.78 (70.1%)
Maximum concentration, mg/L	3.86 (37.7%)	3.36 (65.4%)
Time to C_max_, h	2.7 (50.7%)	2.2 (25.1%)
Half-life, h	12.33 (36.7%)	5.97 (38.8%)
Apparent clearance, mL/min	0.159 (31.7%)	0.339 (59.4%)
Volume of distribution, L	2.83 (21.9%)	2.92 (69.3%)
Dolutegravir metabolic ratio	0.0292 (50%)	0.0667 (66%)

Reported proportions or geometric mean (coefficient of variance). Abbreviation: C_max_, maximum concentration; C_trough_, trough concentration.

### Safety and Efficacy

**Figure 4.**
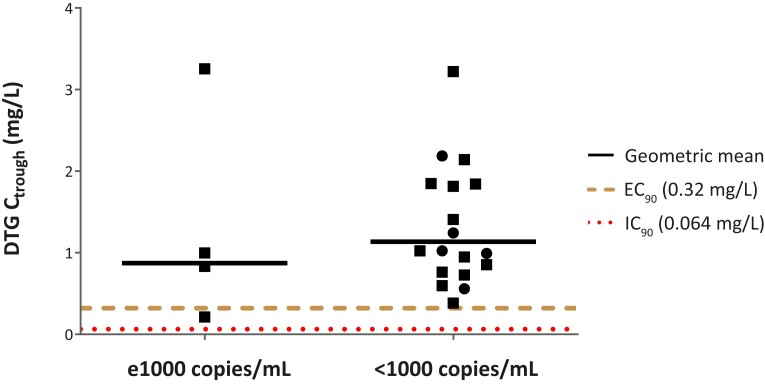
DTG trough concentrations in infants with a human immunodeficiency virus (HIV) viral load >1000 copies/mL on study day 180 versus those with an HIV viral load <1000 copies/mL. Squares represent infants receiving DTG twice-daily dosing with rifampicin; circles indicate infants receiving DTG once-daily dosing without rifampicin. Abbreviations: C_trough_, trough concentration; DTG, dolutegravir; EC_90_, 90% effective concentration; IC_90_, in vitro protein-adjusted 90% maximal inhibitory concentration.

During the 180-day follow-up period, 82 AEs were reported (summarized in Table 3). Four of 21 infants (19%) experienced an AE, of which 2 were SAEs (liver abnormalities) that were potentially related to rifampicin, and 1 of 27 infants (4%) experienced an AE that was possibly related to dolutegravir. All 5 successfully resolved without treatment discontinuation. VL was below 1000 copies/mL in 76% and 100% of infants and undetectable in 35% and 20% of infants with and without rifampicin, respectively. Development of VL during the study is presented in Supplementary Figure 2. Dolutegravir C_trough_ was comparable for infants who had a VL ≥1000 copies/mL versus those with <1000 copies/mL, as shown in Figure 4.

**Table 3.** Descriptive Safety and Efficacy Results

Safety Parameter	Control Arm (n = 6)	Rifampicin Arm (n = 21)	All (n = 27)
Any AE	17 (in 6/6 infants)	65 (in 20/21 infants)	82 (in 26/27 infants)
Any SAE	3 (in 3/6 infants)	23 (in 13/21 infants)	26 (in 16/27 infants)
Drug-related SAEs	0	2 (in 2/21 infants)	2 (in 2/27 infants)
Any drug-related AE	0	5^a^ (in 5/21 infants)	5 (in 5/27 infants)
Grade 3	–	1 (in 1/21 infants)	1 (in 1/27 infants)
Grade 4	–	1 (in 1/21 infants)	1 (in 1/27 infants)
AEs leading to withdrawal	0	0	0

HIV Viral Load Data	Control Arm (n = 5)	Rifampicin Arm (n = 17)	All (n = 22)^b^
Undetectable HIV viral load on study day 180^c^	1 (20%)	6 (35%)	6 (27%)
HIV viral load <1000 copies/mL on study day 180	5 (100%)	13 (76%)	18 (82%)

The safety parameters are reported as number of reported outcomes followed by the percentage of infants with at least 1 reported outcome.

Abbreviations: AE, adverse event; HIV, human immunodeficiency virus; SAE, severe adverse event.

^a^Three alterations in liver function (including 1 grade 4 and 1 grade 3 AE) and 1 skin rash were considered possibly or potentially related to rifampicin, and 1 grade 2 liver alteration was considered possibly related to dolutegravir.

^b^Viral load data at 180 days after enrollment in the main trial were unavailable for 4 infants, and 1 infant was on dolutegravir for less than 120 days.

^c^Detection limit varied between 20 and 150 copies/mL.

## DISCUSSION

To our knowledge, we are the first to report dolutegravir pharmacokinetic data and descriptive safety and efficacy for infants receiving concomitant rifampicin. Our findings showed that BID dosing of dolutegravir in the presence of rifampicin resulted in adequate dolutegravir exposure. The geometric mean C_trough_ was similar in infants receiving dolutegravir OD without rifampicin (1.11 mg/L) and infants receiving dolutegravir BID with rifampicin (1.06 mg/L). Furthermore, the C_trough_ levels in our study population were comparable to C_trough_ levels in adults after administration of 50 mg dolutegravir OD without rifampicin (1.20 mg/L) and in infants receiving dolutegravir OD without rifampicin following WHO weight-band dosing (C_trough_, 1.18 to 1.45 mg/L) [24, 25].

Dolutegravir and rifampicin are widely recognized as the preferred components for treating HIV and TB, respectively, with well-established pharmacokinetics, efficacy, and safety in infants when given in the absence of the other drug [6, 26]. Furthermore, the options for appropriate alternative ART for infants receiving rifampicin-based TB treatment are limited due to suboptimal pharmacokinetic outcomes or limited drug availability [4, 27, 28]. Hence, cotreatment with rifampicin and dolutegravir is preferred for treatment of infants with HIV-associated TB [4, 6]. Moreover, providing dolutegravir-based ART to infants receiving rifampicin also allows for harmonization of preferred ART regimens across all ages.

The appropriate minimum effective concentration of dolutegravir is currently the subject of ongoing debate. The EC_90_, or the concentration of dolutegravir at which 90% of the maximum reduction in VL was observed in a 10-day adult monotherapy study [22], may represent an overestimation of the actual target as various studies have shown adequate treatment outcomes despite C_trough_ that was below the EC_90_ [29]. On the other hand, the IC_90_ (0.064 mg/L) may be an underestimation of the target as it is solely based on in vitro data [23]. The one dolutegravir C_trough_ below EC_90_ in our study was 0.21 mg/L, which is well above the IC_90_, leading to confidence in the conclusion that BID dosing of dolutegravir is sufficient to overcome its interaction with rifampicin in infants with HIV.

While our study showed similar pharmacokinetic profiles in infants compared with older children and adults, the proportion of infants with an undetectable VL after 6 months of follow-up was relatively modest with 35% and 20% of infants with and without rifampicin, respectively. Furthermore, 24% of infants receiving rifampicin and none of the infants without rifampicin had a VL of >1000 copies/mL on day 180 of the study. These findings warrant cautious interpretation given the small sample sizes and the reliance on self-reported dolutegravir treatment adherence by caregivers. It is important to note that time to viral suppression is generally longer in infants and young children on dolutegravir and alternative ART [7]. In different cohorts following CWH who were not acutely ill, similar results were reported, with only 37%, 45%, and 60% of infants achieving virological control after 6, 12, and 24 months of early ART, respectively [30–32]. Response on treatment was deemed unrelated to the pharmacokinetics of dolutegravir in our study as C_trough_ levels were comparable between infants with VL above or below 1000 copies/mL at study visit day 180. The large number of reported AEs and SAEs in this study was deemed related to severe underlying illness that initially led to hospitalization or other treatments. Only 5 AEs, including 2 SAEs (both liver function alterations), were possibly related to rifampicin or dolutegravir. As liver function alterations are not uncommon during first-line TB treatment and all resolved without treatment discontinuation, dolutegravir and rifampicin cotreatment appeared safe in our study population [33].

There is growing interest in continuing OD dosing of dolutegravir in the presence of rifampicin instead of increasing the dolutegravir dose frequency to BID. A recent prospective clinical trial showed promising results for this strategy in adults with HIV–TB coinfection, despite a substantial number of patients (35%) in the OD arm having dolutegravir C_trough_ below the IC_90_ target [34]. Considering that young children generally require a longer time for virological suppression after ART initiation, we advise caution when studying OD dosing of dolutegravir in infants on rifampicin-based TB treatment. On top of that, currently recommended dosages of rifampicin have been reported to result in low exposure (AUC) in infants, and higher dosages may therefore be introduced in the near future [35]. Additionally, studies are exploring the potential use of high-dose rifampicin (up to 35 mg/kg) for children [36, 37]. These developments may result in higher exposure to rifampicin and, as a consequence, a larger decrease in dolutegravir levels, as observed in adults [38].

Dolutegravir’s metabolic ratio was 2.3-fold higher in infants on rifampicin compared with those without rifampicin. This ratio is consistent with an ex vivo study that showed that rifampicin at therapeutic concentrations increases the expression of UGT1A1 in human hepatocytes by 2-fold [39]. Notably, the dolutegravir metabolic ratio was substantially lower in our control arm (0.0292) compared with adult reference values (0.08) [14]. Low activity of UGT could theoretically result in less inducibility of UGT and result in a less pronounced magnitude of the DDI. However, the magnitude of the DDI observed in our study population seems comparable to findings from adult studies that also reported comparable AUC and C_trough_ values between the 2 arms [9, 10]. While the dolutegravir metabolic ratio did not show a significant correlation with age, infants aged <6 months in the rifampicin group appeared to have lower dolutegravir metabolic ratios (see Supplementary Figure 1). This may be because maturation of UGT1A1 primarily occurs within the initial 3 to 6 months of life. Data from a single neonate suggest a very low dolutegravir metabolic ratio in neonates, which is in line with low activity of UGT1A1 in this population [40].

Our study has several limitations including dolutegravir not being administered as an investigational product of the EMPIRICAL trial. Therefore, assessing the relationship between AEs and dolutegravir was not part of the main study aim, and adherence to dolutegravir treatment was self-reported. Nevertheless, dolutegravir being administered as part of standard-of-care practice increases the generalizability of the results to real-world conditions. Furthermore, most infants in our study were considered fed and part of the group that did not have confirmed HIV-TB coinfection, which could have impacted dolutegravir rifampicin exposure, making comparisons with similar DDI studies more complex. However, we believe that this situation reflects real-world practice where infants are fed regularly. Additionally, the sample size in our control arm was small because a large number of infants in the non-TB treatment arms of the main trial received TB treatment following post-randomization clinical or laboratory TB diagnosis. Hence, these infants were ineligible for inclusion in the control arm of our substudy, which complicated the comparative analyses. Despite this, the pharmacokinetic parameters found in our study were comparable to those reported in previous studies evaluating dolutegravir pharmacokinetics in children not receiving rifampicin [21, 25].

In conclusion, consistent with data from older children and adults, BID dosing of dolutegravir in infants receiving rifampicin, following WHO weight-band dosing, resulted in adequate exposure to dolutegravir. These pharmacokinetic data support the use of this dosing regimen in infants with HIV and receiving concomitant rifampicin therapy. Larger studies, including the main EMPIRICAL trial, are expected to assess virological response in infants receiving dolutegravir BID with rifampicin compared with dolutegravir OD without rifampicin.

## Supplementary Data

Supplementary materials are available at *Clinical Infectious Diseases* online. Consisting of data provided by the authors to benefit the reader, the posted materials are not copyedited and are the sole responsibility of the authors, so questions or comments should be addressed to the corresponding author.

## Supplementary Material

ciad656_Supplementary_Data

## Notes

***Acknowledgments.*** The authors thank all of the children and their families who participated in this study.

This project is part of the EDCTP2 program supported by the European Union RIA2017MC-2013.

Members of the EMPIRICAL Clinical Trial Group include the following: Universidade Eduardo Mondlane Faculdade de Medicina, Maputo, Mozambique: Jahit Sacarlal, Muhammad Sidat, Elias Manjate, and Sónia Martins; Hospital Central de Maputo, Maputo, Mozambique: Stella Langa, Natália Nipaco; Hospital Provincial de Matola, Matola, Moza: Sara Machava, Anastância Chirindza; Hospital Geral de Mavalane: Luzidina Martins, Mércia Nhaca; University of Zimbabwe Clinical Research Centre: Kusum J. Nathoo, Moses Chitsamatanga, Ruth Marange, Shepherd Mudzingwa, Dorothy Murungu; University Teaching Hospital, Lusaka, Zambia: Natasha Namuziya, Idah Zulu, Perfect Shankalala, Mulima Mukubesa, Juliet Namwinwa, Chalwe Chibuye, Terence Chipoya, Veronica Mulenga, Bwalya Simunyola, John Tembo; Arthur Davidson Children’s Hospital, Ndola, Zambia: Muleya Inambao, Salome Chitondo, Wyclef Mumba, Endreen Mankushe, Henry Musukwa, Davies Sondashi; China Uganda Friendship Hospital Naguru, Kampala, Uganda: Albert Kamugisha, Karen Econi; Makerere University Lung Institute, Kampala, Uganda: Andrew Kiggwe, Judith Beinomugisha, Sharafat Nkinzi, Lawrence Kakooza, Henriator Namisanvu, Nancy Lajara Mark, Josam Thembo Mwesige, Ivan Segawa, Joseph Ssessanga, Paul Mbavu; Infectious Diseases Institute Laboratory, Makerere University, Kampala, Uganda: Bosco Kafufu; Mbarara Regional Referral Hospital, Mbarara, Uganda: Denis Nansera, Elizabeth Najjingo, Bashira T. Mbabazi; Joint Clinical Research Centre, Regional Centre of Excellence, Mbarara, Uganda: Abbas Lugemwa, Mariam Kasozi, Rogers Ankunda; Pediatric Unit for Research and Clinical Trials, Hospital 12 de Octubre Health Research Institute (i + 12), Biomedical Foundation of Hospital Universitario 12 de Octubre (FIB-H12O), Madrid, Spain: Lilit Manukyan.

***Data sharing statement.*** The data that support the findings of this study are available from the corresponding authors upon reasonable request.

Financial support. This project is funded by the European and Developing Countries Clinical Trials Partnership (EDCTP2) program supported by the European Union (RIA2017MC-2013). U. C. and W. C. B. report involvement in 2 EDCTP-funded studies in Mozambique (EMPIRICAL and Impact of oral step-down to amoxicillin or co-amoxiclav and of duration of antibiotic therapy on effectiveness, safety and selection of antibiotic resistance in severe childhood community-acquired pneumonia (PediCAP) trials).
