# Supplementary material for: Twice-Daily Dosing of Dolutegravir in Infants on Rifampicin Treatment: A Pharmacokinetic Substudy of the EMPIRICAL Trial
Source: Clin Infect Dis. 2023 Oct 26;78(3):702–10. doi: 10.1093/cid/ciad656 (PMC10954323; doi:10.1093/cid/ciad656)
Supplement: ciad656_Supplementary_Data [file ciad656_supplementary_data.docx]

Supplementary files

**Twice-daily dosing of dolutegravir in infants on rifampicin treatment: a pharmacokinetic substudy of the EMPIRICAL trial**

*Authors:* Tom G. Jacobs, Vivian Mumbiro, Uneisse Cassia, Kevin Zimba, Damalie Nalwanga, Alvaro Ballesteros, Sara Domínguez-Rodríguez, Alfredo Tagarro, Lola Madrid, Constantine Mutata, Moses Chitsamatanga, Mutsa Bwakura-Dangarembizi, Alfeu Passanduca, W. Chris Buck, Bwendo Nduna, Chishala Chabala, Elizabeth Najjingo, Victor Musiime, Cinta Moraleda, Angela Colbers, Hilda A. Mujuru, Pablo Rojo, David M. Burger, on behalf of the EMPIRICAL clinical trial group

Supplementary file 1 – Correlations between pharmacokinetic and demographic parameters

Supplementary file 2 – HIV viral load development over time

# Supplementary file 1 – Correlations between pharmacokinetic and demographic parameters


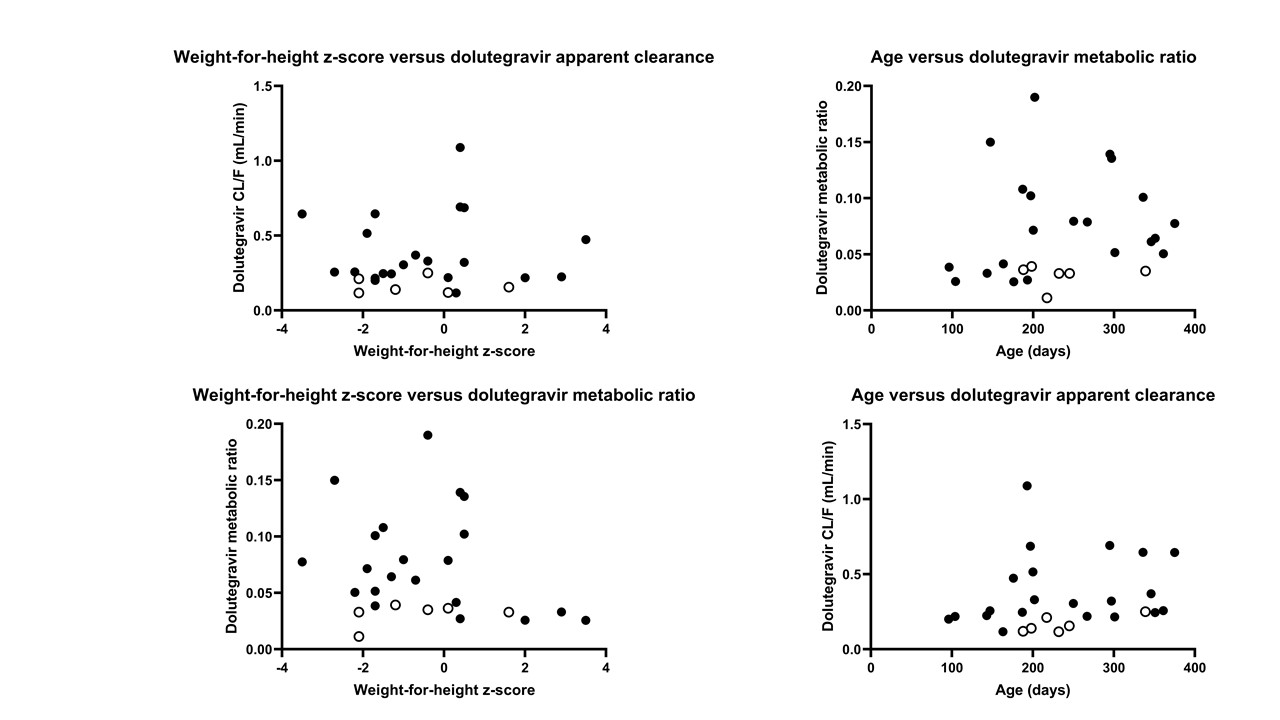


**Figure S1** Scatterplots displaying correlations between dolutegravir apparent clearance and metabolic ratio with age and weight-for-height. The bold dots represent infants receiving dolutegravir BID with rifampicin and the open dots represent infants receiving dolutegravir OD without rifampicin.

# Supplementary file 2 – HIV viral load development over time


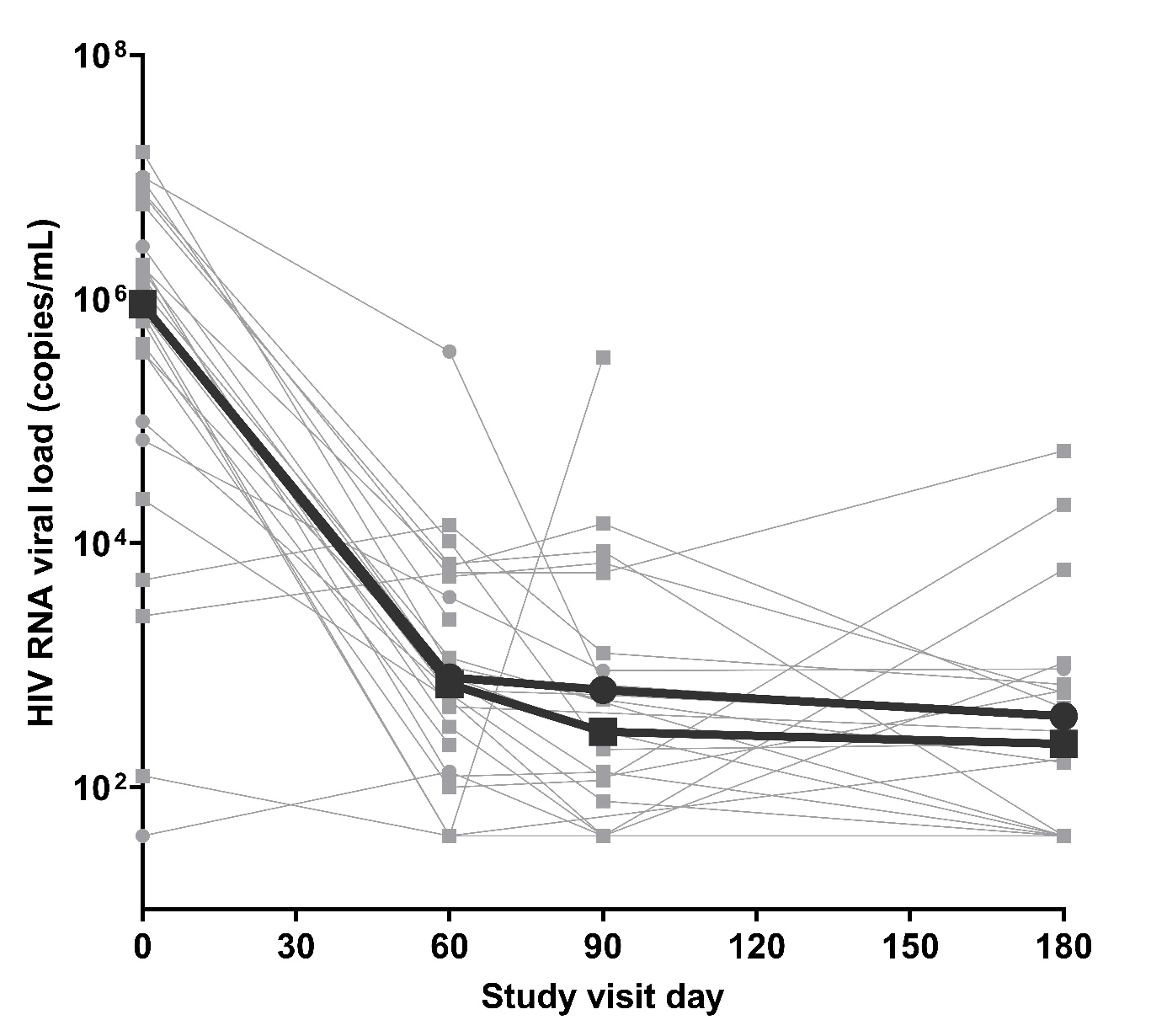


**Figure S2** The development of HIV viral load over time in all study participants. The squares represent infants receiving dolutegravir BID with rifampicin and the dots represent infants receiving dolutegravir OD without rifampicin. The bold lines represent the median viral load per study arm. The x-axis includes the number of days after enrolment in the main EMPIRICAL trial. Of note, all children were initiated on ART prior to day 30 of the main trial.
